# Early Mobility in the Hospital: Lessons Learned from the STRIDE Program

**DOI:** 10.3390/geriatrics3040061

**Published:** 2018-09-26

**Authors:** Susan Nicole Hastings, Ashley L. Choate, Elizabeth P. Mahanna, Theresa A. Floegel, Kelli D. Allen, Courtney H. Van Houtven, Virginia Wang

**Affiliations:** 1 Center of Innovation for Health Services Research in Primary Care, Durham VA Health Care System, Durham, NC 27705, USA; ashley.choate@va.gov (A.L.C.); elizabeth.mahanna@va.gov (E.P.M.); kelli.allen@va.gov (K.D.A.); courtney.vanhoutven@va.gov (C.H.V.H.); virginia.wang@duke.edu (V.W.); 2 Department of Medicine, Duke University School of Medicine, Durham, NC 27710, USA; 3 Center for the Study of Aging and Human Development, Duke University School of Medicine, Durham, NC 27710, USA; 4 Geriatrics Research, Education, and Clinical Center, Durham VA Health Care System, Durham, NC 27705, USA; 5 College of Nursing, East Carolina University, Greenville, NC 27858, USA; floegelt16@ecu.edu; 6 Department of Medicine & Thurston Arthritis Research Center, University of North Carolina at Chapel Hill, Chapel Hill, NC 27599, USA; 7 Duke Department of Population Health Sciences, Duke University School of Medicine, Durham, NC 27701, USA

**Keywords:** mobility, hospitalization, older adults, implementation

## Abstract

Immobility during hospitalization is widely recognized as a contributor to deconditioning, functional loss, and increased need for institutional post-acute care. Several studies have demonstrated that inpatient walking programs can mitigate some of these negative outcomes, yet hospital mobility programs are not widely available in U.S. hospitals. STRIDE (assiSTed eaRly mobIlity for hospitalizeD older vEterans) is a supervised walking program for hospitalized older adults that fills this important gap in clinical care. This paper describes how STRIDE works and how it is being disseminated to other hospitals using the Replicating Effective Programs (REP) framework. Guided by REP, we define core components of the program and areas where the program can be tailored to better fit the needs and local conditions of its new context (hospital). We describe key adaptations made by four hospitals who have implemented the STRIDE program and discuss lessons learned for successful implementation of hospital mobility programs.

## 1. Introduction

Hospitalized adult patients spend 95% of their time in beds or chairs [1,2,3]. Patient immobility during a hospitalization is linked to pressure ulcers, venous stasis, decreased cardiac output, longer hospital length of stay (LOS), and an increased risk of hospital-associated pneumonia [1,4]. Prolonged hospital immobility is also associated with significant reductions in muscle mass and strength, leading to functional decline [5]. This deconditioning is one of the most common reasons for delayed discharge [6,7], and is associated with early readmission [8]. Older hospitalized adults are at even greater risk for reduced mobility and functional decline due to aging processes and higher prevalence of chronic diseases. Approximately 30 to 60% of older adults lose some degree of functional independence during a hospital stay [9] which may subsequently lead to the requirement for post-acute and long-term institutionalization [6].

Many factors may contribute to the negative outcomes related to low patient mobility, including patient-related and treatment factors [10]. The most common barriers to mobility cited by older hospital patients are feelings of weakness, pain, fatigue, and having an intravenous or urinary catheter. Institutional and personal factors may also influence patient mobility. Specifically, nursing staff report insufficient resources (e.g., assistive personnel) [10,11] and high workloads for nurses [12] as barriers to mobilizing patients. A lack of mobility assistive devices in hospital rooms and hallways (e.g., bedside commode, walker) were also cited by both patients and staff as inhibiting mobility for older adults [10].

Interventions to systematically promote early, safe mobility in older hospitalized patients—designed to address clinical and logistical challenges of promoting mobility—are needed to avert the negative and costly consequences of immobility during inpatient stays. Previous studies have demonstrated the potential of inpatient walking to prevent declines in mobility during hospital stays and to reduce hospital lengths of stay [13,14,15,16,17,18,19]. For example, the MOVE ON (Mobilization of Vulnerable Elders in Ontario) early mobility program for older, hospitalized patients in Ontario, Canada showed a decrease in median length of stay (−6.1 days) after implementation of the program in 11 participating hospitals [17]. However, hospital mobility programs have not been widely adopted in U.S. hospitals. Previous studies evaluating inpatient mobility programs typically have focused on the effect of the program itself, but have not addressed the efforts required to successfully plan and implement the program. This paper describes the ongoing implementation of STRIDE (assiSTed eaRly mobIlity for hospitalizeD older vEterans), a supervised walking program for hospitalized older adults that fills this important gap in clinical care. We describe features of the STRIDE program and how it is being disseminated to other hospitals using the Replicating Effective Programs (REP) framework. Guided by REP, we define core components of the STRIDE program and areas where the program can be tailored to better fit the needs and local conditions of different hospitals and/or health systems. We describe key adaptations made by four hospitals who have implemented the STRIDE program and discuss lessons learned for successful implementation of hospital mobility programs. 

## 2. Assisted Early Mobility for Hospitalized Older Veterans: The STRIDE Program

STRIDE (assiSTed eaRly mobIlity for hospitalizeD older vEterans) is a supervised walking program for hospitalized older adults focused on maintaining musculoskeletal strength and mobility during hospitalization, a highly vulnerable time for development of disability. It was adapted from a mobility program, studied in a randomized controlled trial in three non-Veterans Health Administration (non-VA) hospitals, that led to reduced hospital lengths of stay [20]. Originally developed and tested at the Durham VA Health Care System (VAHCS) as a clinical demonstration program in 2012, STRIDE was designed for patients aged >60 years, the demographic group most susceptible to the negative consequences of immobility in the hospital [21]. Durham’s STRIDE program consists of a one-time gait and balance assessment conducted by a physical therapist, followed by daily supervised walks (median duration 10 min) led by a recreation therapy assistant for the duration of the hospital stay. Initial program evaluation found that STRIDE participants were more likely to be discharged to home than skilled nursing or rehabilitation compared to clinically similar patients receiving usual care (92% vs. 74%, *p* = 0.007) [22]. Based on the cumulative evidence on early mobility and the positive results from the STRIDE demonstration, the Durham VAHCS established STRIDE as a permanent clinical service in 2013 [23].

## 3. Descriptive Evaluation of STRIDE Expansion

Initial positive results suggested that STRIDE had the potential to become a system-wide approach to address hospital-associated disability in the VA; however, differences in resources, staffing, and priorities among other conditions in VA facilities presented barriers to successful implementation at other sites. Therefore in 2016 we launched an ongoing project, Optimizing Function and Independence Quality Enhancement Research Initiative (known as “Function QUERI”) to implement STRIDE in eight additional VA hospitals [24] in 2017–2019. 

### 3.1. Methods and Data Sources

We used VA administrative data sources [25,26] to describe site characteristics (Table 1). Data were also derived from detailed notes from a series of calls conducted by a facilitator with key personnel at each expansion site. Key personnel included staff members who would be implementing the program, referring providers, staff conducting the gait assessments or supervised walking, and ward and service line leadership. On each of the calls (described in more detail in Section 3.2), a notetaker recorded the content and summarized key points afterwards. In addition, pre-implementation interviews were conducted with key personnel at each site (*n* = 35 interviews) and summarized into case summaries for each of the four sites. Data from all sources (administrative data, call notes, case summaries, and provider interviews) were combined in a matrix to view emergent patterns that served as the basis for program characteristics and REP adaptations described in Section 3.3 and Table 2.

### 3.2. Participating Sites

One of the goals of Function QUERI is to study the process of starting a STRIDE program so that these lessons can be applied in broader efforts to promote hospital mobility programs. Eight VA hospitals volunteered to begin STRIDE programs at their sites and were randomized to begin at a specific time [24]. To date, four of eight have initiated their programs (hereafter referred to as expansion sites). Characteristics of the original and four expansion sites (to date) are presented in Table 1.

### 3.3. Implementation Framework: Replicating Effective Programs

A one-size-fits-all approach is rarely effective for addressing the barriers of implementing a program in multiple new settings [29]; therefore, the Function QUERI team is using the Replicating Effective Programs (REP) framework to facilitate expansion of the program at each expansion site. REP consists of a series of activities to support implementation of the core elements of a program while allowing space for stakeholder input and flexibility to modify the program to site specific resources and patient needs [29,30,31,32]. There are four key phases in the REP framework that standardize activities for the rollout of a new clinical program: (1) Pre-Conditions; (2) Pre-Implementation; (3) Implementation; and (4) Maintenance and Evolution [29]. The REP framework provides a standardized guide of intervention protocol, training, and technical assistance for sites to strategically customize the STRIDE program to fit their local settings while addressing barriers to implementation. STRIDE expansion sites participated in REP activities through a series of six scheduled calls and one in-person site visit by the Function QUERI team over the course of three months prior to program launch. After STRIDE launch, expansion sites participated in five additional scheduled calls with the Function QUERI team to review data, identify barriers to implementation, and develop strategies to overcome those barriers.

#### 3.3.1. Pre-Conditions Phase

All expansion sites identified a need to improve mobility of their hospitalized patients and selected the STRIDE program as a means to address this clinical problem. The Pre-Conditions phase of REP focuses on leveraging the pre-existing resources in the health care system essential to implementing a new clinical program. Key tasks include identification of local champions or advocates who are essential in establishing widespread buy-in and ensuring the program fits pre-existing culture, local programmatic goals, and resources [33]. Each expansion site staff identified a primary coordinator for program implementation. The Function QUERI implementation team worked closely with local champions to identify barriers and facilitators to program implementation specific to each expansion site, and these are used to inform decisions such as which service lines (e.g., rehabilitation and nursing) and leaders to include in their implementation teams for rolling out the STRIDE program.

#### 3.3.2. Pre-Implementation Phase

The Pre-Implementation phase focuses on preparation for program implementation and obtaining broad buy-in for the new clinical program. The key tasks include identifying key stakeholders and tailoring the program materials to fit the local context. Each expansion site identified key stakeholders to serve on the local STRIDE implementation team, including VA Medical Center leadership, service-line chiefs, and front-line staff. They identified champions from the services lines with key roles in program implementation. Topics covered during pre-implementation phase calls including describing the intervention protocol (e.g., standardized program materials, procedures, and competencies) and providing technical assistance to guide sites on options for customization and prepare for program launch. During this phase, the expansion site teams discussed their site’s staffing model, patient eligibility criteria, and target distance and time for daily patient walks. The Function QUERI team conducted a site visit prior to program launch to provide on-site technical assistance (e.g., implementing/tailoring program documentation) and market the program to local leadership and front-line staff.

#### 3.3.3. Implementation Phase

The Implementation phase begins with program launch and includes ongoing training and technical assistance, program promotion to increase buy-in among front-line staff and leadership, and process monitoring to ensure program feasibility and fidelity to the core components of the STRIDE program. The local STRIDE implementation team at expansion sites received weekly reports of program activity to facilitate ongoing improvements. For example, reports included counts of STRIDE consults and percentage of hospital days that STRIDE patients had at least one documented walk. Data review helped to identify barriers to successful implementation and promote discussion of strategies to overcome these. For example, if the number of consults at an expansion site was low, the Function QUERI and expansion site teams discussed strategies for improvement, such as increased marketing of the program to residents and other medical staff.

#### 3.3.4. Maintenance and Evolution Phase

The final Maintenance and Evolution phase involves compiling program results and reporting to leadership and other relevant stakeholders, as well as using process and outcome results to further refine program implementation for other sites. For example, some sites are reporting data to leadership on veteran satisfaction with the program and relevant performance metrics (e.g., length of stay) on wards which implemented STRIDE. The final phase includes continued technical assistance, continued reports of program activity, and ongoing consultation about program implementation and evolution to ensure sustainability. The Function QUERI team is refining a STRIDE implementation package, with the sites’ input, in preparation for wider-scale dissemination.

### 3.4. Core Components and Adaptations

The Function QUERI team viewed the REP Framework as ideal for the rollout of STRIDE because the framework provides a standard structure for specifying core elements of the program while allowing sites flexibility to determine which components can be adapted to local settings. Essential core elements of STRIDE were defined as:

(1) Proactive, no baseline functional deficits required
(2) Early enrollment, ideally within 24 h of admission
(3) Supervised walking, up to 20 min daily until discharge

The STRIDE team encouraged program adaptations in the areas of how to identify patients for the program (referral model) and the role of the staff who would conduct the assessment and supervise the walks (staffing model). Table 2 provides examples of how the original and expansion sites designed their STRIDE programs. STRIDE coordinators were all clinicians (three physicians (MD) and two physical therapists (PTs)). Three sites hired additional staff to implement the program while two implemented the program using existing staffing resources. All sites used PTs to conduct initial gait assessments but a variety of professional roles were engaged to supervise walks, including recreation therapy assistants, certified nursing assistants (CNAs), PT assistants, kinesthiotherapists (KTs) and nurses.

## 4. Lessons Learned

Function QUERI’s experience with STRIDE implementation at four expansion sites has resulted in several lessons learned that are relevant for future dissemination efforts and also for individual hospitals interested in starting a mobility program.

### 4.1. Flexible Models of STRIDE Delivery

Due to competing demands on staff time, flexibility around the STRIDE staffing model is important. Decisions about which staff roles are best suited to supervise walks and how these are structured (e.g., collateral duties vs. dedicated STRIDE personnel), should be made collaboratively with representation from all key stakeholders. Leadership buy-in and front-line clinical champions have been critical to initiate the program and sustain the weekly consults, gait assessments, and walking sessions. Because most models have incorporated physical therapists to conduct the gait assessment and nurses or CNAs to supervise the walks, effective interdisciplinary communication between those two professional groups is particularly vital. Coordination with other groups charged with improving quality and safety in the hospital, such as fall prevention committees and safe patient handling and mobility coordinators is also key to ensure programmatic alignment. 

### 4.2. Technical/Logistical Support

From a training and documentation perspective, providing competency checklists to the facility to adapt for staff training and modifiable electronic health record templates to document gait assessments and walks in a standardized, reportable manner have helped save time and effort for facilities and enhanced the ability of sites to track their progress. The electronic health record templates have included standardized data that can be extracted as structured text fields such as patient enrollment in the program, walk distance and time, and how the patient felt after the walk. The ability to compile these data periodically has helped sites to monitor their progress, troubleshoot issues that arise, and begin to build a case with leadership for program growth and sustainability. For Function QUERI, it is essential that all hospitals use standardized data collection templates to facilitate combining data across sites. For implementation support provided outside of a study context we anticipate that fewer calls would be required to meet the data needs of individual sites.

### 4.3. Culture Change

Through interviews with the expansion sites’ staff and leadership, we discovered that service line leadership and other key stakeholders often viewed the STRIDE program as a platform for broader culture change around patient mobility. We found that site personnel usually recognized the value of patient mobility but needed the presence of an evidence-based, implementable program to “jumpstart” change in that area and address the biases of some staff or facility procedures that were hindering mobility. For example, two sites changed activity orders to reduce bedrest orders and encourage ambulation. One site developed a ‘huddle board’ at the nursing station to communicate about patient mobility and STRIDE consults. 

### 4.4. Limitations

STRIDE was based on a hospital mobility intervention proven effective in a randomized controlled trial [20], however to date, evidence of effectiveness in the VA has only been examined with a quasi-experimental design [22]. Our ongoing Function QUERI stepped wedge, cluster randomized trial [24] is designed to provide additional high-quality evidence about the impact on patient outcomes and utilization in the VA. Formal qualitative analyses of data collected in provider interviews with expansion site personnel will provide additional insights into STRIDE implementation.

### 4.5. Future Research Directions

Combining data from all eight expansion sites that ultimately implement STRIDE as part of Function QUERI will expand our understanding of the impact of hospital mobility programs on patient and system-level outcomes. Our implementation evaluation will focus on the roles of teams in successfully initiating and sustaining STRIDE, and whether a teamwork training intervention can enhance implementation of new clinical programs. Future research should address whether there are differential effects of hospital mobility programs according to patient characteristics (e.g., baseline physical function or disease states such as congestive heart failure) as well as whether extending interventions into the home setting after discharge may be beneficial. Direct patient feedback from pedometers or other activity trackers and/or motivational incentives may offer a means of enhancing activity during hospitalization between STRIDE walks.

## 5. Conclusions

Although the hazards of immobility during hospitalization have been long-known, sustained solutions to this problem have been elusive. STRIDE offers a promising approach for hospitals to support increased mobility in their patients, thus supporting better patient outcomes and reduced need for post-acute institutional care. As part of the final REP phase (Maintenance and Evolution), we plan to package a clinical program guide to assist with wider dissemination of the STRIDE program to sites outside of the Function QUERI study. Using the REP framework processes may help individual sites considering hospital mobility programs to tailor their program to best meet their needs, while ensuring that key components shown previously to be effective are retained.

## Figures and Tables

**Table 1:** Site characteristics.

Site Characteristic	Original Site	Expansion Site 1	Expansion Site 2	Expansion Site 3	Expansion Site 4
Location	SE	SE	SE	MW	MW
Inpatient medical or surgical beds	271	466	109	361	51
Complexity level	1A	1A	1C	1A	1C
Star rating	3	3	3	5	3

SE = Southeast United States; MW = Midwest United States. Complexity level: A rating that divides veteran’s health facilities (VA) facilities into five levels (1A, 1B, 1C, 2, 3; highest to lowest), based on levels of patient volume and risk, teaching and research, intensive care units, and physician specialist staffing [27]. Star rating: A composite indicator of hospital performance relative to other VA medical centers (scale 1 to 5; low to high) [28].

**Table 2:** STRIDE program characteristics.

Program Characteristic	Original Site	Expansion Site 1	Expansion Site 2	Expansion Site 3	Expansion Site 4
STRIDE coordinator role	PT	MD	MD	MD	PT
Hired new staff for STRIDE program	Yes	No	No	Yes	Yes
Initial number of STRIDE wards	2	2	3	2	1
Staff role for referrals to STRIDE Program	Medical providers, PTs	Medical providers, PTs	ACE Team, Medical providers	Medical providers, PTs	PTs
Staff role for gait assessment	PT	PT	PT	PT	PT
Staff role for walking (alternates)	Recreation therapy assistant	CNA	CNA	PT assistant (nurses)	PT assistant (KTs, nurses)

PT = physical therapist. MD = physician. ACE = Acute Care for the Elderly; an interdisciplinary care program focusing on frail, aged inpatients at elevated risk for decline, disorientation, delirium, and extended length of stay. CNA = certified nursing assistant. KT = kinesthiotherapist. Medical providers = MD, advanced practice nurse or physician’s assistant. STRIDE = assiSTed eaRly mobIlity for hospitalizeD older vEterans.
